# Silver Carp (*Hypophthalmichthys molitrix*) Scale Collagen Peptides-1 (SCPs1) Inhibit Melanogenesis through Downregulation of the cAMP-CREB Signaling Pathway

**DOI:** 10.3390/nu15112449

**Published:** 2023-05-24

**Authors:** Hai-Lan Li, Mei-Jin Li, Guang-Quan Xiong, Jun Cai, Tao Liao, Xiao-Yan Zu

**Affiliations:** 1 Key Laboratory of Cold Chain Logistics Technology for Agro-Product, Ministry of Agriculture and Rural Affairs, Institute of Agro-Products Processing and Nuclear Agricultural Technology, Hubei Academy of Agricultural Sciences, Wuhan 430064, China; 2 Key Laboratory of Fermentation Engineering, Ministry of Education, Hubei Provincial Cooperative Innovation Center of Industrial Fermentation, Hubei Key Laboratory of Industrial Microbiology, Hubei University of Technology, Wuhan 430068, China

**Keywords:** scale collagen peptides-1, melanogenesis, cAMP-CREB, whitening

## Abstract

The mechanism of silver carp scale collagen peptides (SCPs1) on melanogenesis and its mechanism of action were examined in mouse melanoma cells (B16). The cell viability and effects of SCPs1 on intracellular tyrosinase (TYR) activity and melanin, reactive oxygen species (ROS), glutathione (GSH) and cyclic adenosine monophosphate (cAMP) content were examined. The regulatory mechanism of SCPs1 on the cAMP response element-binding protein (CREB) signaling pathway was analyzed. The cell viability of the SCPs1 group was >80% (0.01–1 mg/mL) and the inhibitory rate of SCPs1 on B16 cell melanin increased in a dose-dependent manner. The highest inhibitory rate of SCPs1 on melanin content reaching 80.24%. SCPs1 significantly increased the GSH content and decreased the tyrosinase activity, as well as the content of ROS and cAMP. Western blot analysis showed that SCPs1 significantly inhibited melanocortin-1 receptor (MC1R) expression and CREB phosphorylation in the cAMP-CREB signaling pathway, leading to downregulation of microphthalmia-associated transcription factor (MITF) and the expression of TYR, TYR-related protein-1 (TRP-1) and TRP-2. SCPs1 also inhibited the expression of MC1R, MITF, TYR, TRP-1 and TRP-2 at the transcriptional level. Taken together, SCPs1 inhibited melanin synthesis through the downregulation of the cAMP-CREB signaling pathway. Fish-derived collagen peptides could potentially be applied in skin whitening products.

## 1. Introduction

Melanin has an important effect on human skin color, protecting the skin from ultraviolet (UV) damage [1]. However, overexpression of melanin can generate darkening of the skin tone, pigmentation disorders, melasma, age spots, freckles, hyperpigmentation and other problems [2]. Melanin synthesis is a multi-stage enzymatic process. Tyrosinase (TYR), TYR-related protein-1 (TRP-1) and TYR-related protein-2 (TRP-2) regulated the melanogenesis, with TYR being the key rate-limiting enzyme of the melanogenesis pathway. The microphthalmia-associated transcription factor (MITF) is important in the regulation of intracellular TYR and related enzymes because it specifically binds to the “M-box” structure [3]. The cAMP-CREB pathway is one of the important pathways that regulates MITF and melanogenesis [4]. As an important physiological stimulus of the melanogenesis pathway, ultraviolet light (UV) activates the binding of α-melanocyte-stimulating hormone (α-MSH) to melanocortin-1 receptor (MC1R), which sequentially activates the production of cyclic adenosine monophosphate (cAMP), cAMP-dependent protein kinase A (PKA) and phosphorylation of cAMP response element binding protein (CREB), ultimately upregulating MITF gene expression, leading to increased expression of TYR and other melanogenic enzymes [5]. Xiaohong An et al. showed that pterostilbene inhibited the expression of genes such as MITF and TYR through downregulation of the cAMP-CREB pathway, resulting in the inhibition of melanin synthesis [6]. Xiangna Zhang et al. studied the effect of tea catechins on B16F10 melanoma cells and showed that tea catechins significantly inhibited melanin production by downregulating the cAMP-CREB signaling pathway [7]. Yuanyuan Liu et al. showed that the zebrafish phosvitin-derived peptide Pt5 inhibited melanin synthesis through the cAMP signaling pathway, but it was not regulated by other pathways, such as Wnt/β-catenin [8].

Silver carp (*Hypophthalmichthys molitrix*) is a fast-growing, economical fish that is rich in nutritional value [9]. Fish scales are one of the major byproducts that are produced during the processing of fish products, yet their rich collagen content is rarely utilized, leading to a loss of the associated potential economic value [10]. The enzymatic hydrolysis of collagen to obtain collagen peptides with different biological activities is an effective way to efficiently utilize this byproduct [11]. Natural skin whitening ingredients are more acceptable than chemical ingredients [12]. Therefore, fish-derived collagen peptides, as naturally derived ingredients, are gradually becoming a hot topic for research owing to their whitening activity. At present, research on the whitening activity of fish-derived collagen peptides is mainly focused on the preliminary in vitro experimental stage, and relatively little research has been conducted on its related mechanisms and signaling pathways.

In this work, mouse melanoma cells (B16) were examined with different concentrations of SCPs1, and cell viability was detected by the CCK-8 method; after selecting the appropriate concentration of SCPs1, intracellular tyrosinase activity, melanin, reactive oxygen species (ROS), glutathione (GSH) and cAMP content were measured to analyze the melanin inhibitory mechanism of SCPs1 on B16 cells. Western blot and real-time fluorescence quantitative PCR (RT-PCR) techniques were performed to further elucidate the regulatory mechanism of SCPs1 on the intracellular cAMP-CREB signaling pathway. This study provides data to support the potential application of fish-derived collagen peptides in natural whitening products.

## 2. Materials and Methods

### 2.1. Materials and Reagents

Sliver carp scales were provided by Liangzihu Aquatic products Processing Co., Ltd. (Wuhan, China). Pepsin (1200 U/g) was provided from Sinopharm Chemical Reagent Co., Ltd. (Shanghai, China). Commercially available tilapia scales collagen peptides (CAPs) (Mw = 600–1500 Da) were purchased from Hainan Huayan Collagen Technology Co., Ltd. (Hainan, China). Mouse melanoma cells (B16) were purchased from iCell Bioscience Inc. (Shanghai, China). Fetal bovine serum and trypsin-EDTA were purchased from Wuhan Procell Life Science & Technology Co., Ltd. (Wuhan, China). PBS and DMSO were purchased from Gino Biomedical Technology Co., Ltd. (Zhejiang, China). CCK-8 was purchased from Beyotime Biotechnology Co., Ltd. (Shanghai, China). The ROS assay kit and reduced glutathione (GSH) assay kit were purchased from Nanjing Jiancheng Technology Co., Ltd. (Nanjing, China). The cAMP ELISA Kit, EntiLink™ 1st Strand cDNA Synthesis Super Mix and EnTurbo™ SYBR Green PCR SuperMix were purchased from ELK Biotechnology Co., Ltd. (Wuhan, China). Antibody for β-Actin was supplied by Beijing Tianderui Biotechnology Co., Ltd. (Beijing, China). Antibodies for TYR, TRP-1 and MC1R were obtained from Abcam Biotechnology Co., Ltd. (Hangzhou, China). Antibodies for TRP-2, MITF were purchased from Proteintech Group, Inc. (Wuhan, China). Antibodies for p-CREB, CREB were purchased from Cell Signaling Technology, Inc. (Danvers, MA, USA). The BCA protein assay kit, ECL chemiluminescent substrate assay kit, RIPA lysis buffer, primary antibody and secondary antibody diluents were purchased from Wuhan ASPEN Biotechnology Co., Ltd. (Wuhan, China).

### 2.2. Preparation of SCPs1

Scales were mixed with 4% (*w*/*v*) hydrochloric acid solution according to the solid–liquid ratio of 1:50 and decalcified with a stirrer (JJ-1/100 W, Changzhou Zhiborui Instrument Manufacturing Co., Ltd. (Changzhou, China)) for 6 h. The decalcified scales were hydrolyzed using pepsin to obtain collagen peptides, with enzymatic addition amount 16.1%, solid-to-liquid ratio 1:15.6 and hydrolysis time 4.9 h. Following a 20 min boiling water bath treatment and a 25 min centrifugation (7155× *g* at 18 °C; TGL-24MC, Pingfan Instrument Co., Ltd. (Changsha, China)), the supernatant was collected and the pH was adjusted to neutral. The product was then suctioned and separated by ultrafiltration membrane (S-UF10, Langjimofenli Equipment Engineering Co., Ltd. (Shanghai, China)) with a molecular weight cut-off of 10 kDa. The SCPs1 of the <10 kDa component filtrate was collected. The <10 kDa component was desalinated using a disposable dialysis bag with a molecular weight cut-off of 100 Da, vacuum freeze dried and stored at −20 °C for subsequent use. Based on MALDI-TOF/MS (Bruker ultraflextreme, Bruker Scientific Technology Co., Ltd., Billerica, MA, USA), the main distribution of SCPs1 molecular weight was determined to be in the range of 6096.68–9513.70 Da.

### 2.3. Determination of B16 Cell Viability

Using the technique by Huajin Zeng as a reference with slight modifications [13], logarithmic growth-phase B16 cells were hydrolyzed with trypsin, prepared in a cell suspension with a concentration of 1 × 10^5^ cells/mL, inoculated into a 96-well plate with 1×10^4^ cells/well (100 μL per well) and incubated at 37 °C and 5% CO_2_ (SCO6WE-2, Sheldon Manufacturing Inc., Cornelius, OR, USA) until cell attachment occurred. The culture medium of each group was replaced with serum-free culture medium containing 1% BSA (100 μL per well), where cells were starved for 12 h. The culture medium of each group was replaced with 100 μL of SCPs1 solution for the experimental group and CAPs solution for the active control group, which were diluted by the culture medium at concentrations of 0.01, 0.1, 0.5, 1.0 and 10.0 mg/mL and incubated for 48 h. All wells received an addition of 10 μL CCK-8 solution and were incubated for 1–4 h. A microplate reader (DR-200Bs, Hiwell Diatek Instruments Co., Ltd., Wuxi, China) was used to detect absorbance value A at 450 nm, taking solvent-treated cells to be the control group and regarding empty wells as “blank”. The following Equation (1) was used to calculate cell viability:

(1) $$\mathrm{Cell}\;\mathrm{viability}\%=\frac{A\prime \;\mathrm{Experimental}\;\mathrm{group}-A\prime \;\mathrm{blank}}{A\prime \;\mathrm{Control}\;\mathrm{group}-A\prime \;\mathrm{blank}}\times 100\%$$

### 2.4. Determination of Melanin Content

The cell density was changed to 1 × 10^5^ cells/well, and cells were added to a 6-well plate, with each well inoculated with 2 mL of cell suspension. The resulting attached cells were subjected to cell treatment and culture conditions similar to those outlined previously. The concentrations of the experimental group SCPs1 solution and active control group CAPs solution were set at 0.01, 0.05, 0.1, 0.5 and 1.0 mg/mL, while the solution of the blank control group was replaced with an equal volume of culture medium. There were three replicate wells created for every concentration. Cells were harvested after 48 h of incubation and quantified after counting. The melanin content was determined using the technique by Hideya Ando et al. [14]. The cells were lysed with 1M NaOH solution containing 10% DMSO, underwent ultrasound-assisted degradation for 30 min, were immersed in a water bath at 90 °C for 2 h and were centrifuged at 1000× *g* for 15 min. The OD value was measured at 450 nm using a microplate reader, the relative melanin content of the cells was calculated according to Equation (2) and cell morphology was observed.

(2) $$\mathrm{Relative}\;\mathrm{melanin}\;\mathrm{content}\%=\frac{\mathrm{Treatment}\;\mathrm{group}\;{\mathrm{OD}}_{450}}{\mathrm{Blank}\;\mathrm{control}\;\mathrm{group}\;{\mathrm{OD}}_{450}}\times 100\%$$

### 2.5. Measurement of TYR Activity and ROS, GSH and cAMP Content

Cell inoculation density, treatment, culture conditions and sample concentrations were as described previously, while the culture medium in the blank control group was replaced with an equal volume of culture medium. Three replicate wells were set up for each concentration, and cells were harvested after 48 h of incubation and quantified via counting. The cells were washed twice with pH 6.8 phosphate buffer (0.1 M), and 300 μL of buffer containing 5% (*v*/*v*) TritonX-100 was added to each well. After repeated freeze–thawing and ultrasound-assisted degradation in an ice bath, 200 μL of the supernatant was prewarmed at 37 °C for 10 min, and 500 μL of 0.1% L-DOPA solution was added immediately. After shaking, the absorbance value A was read at 475 nm wavelength on a UV spectrophotometer (UH5300, Hitachi Co., Ltd., Tokyo, Japan) at 0 and 30 min, and the relative cellular TYR activity was calculated according to Equation (3).

(3) $$\mathrm{TYR}\;\mathrm{relative}\;\mathrm{activity}\;\%=\frac{A\prime 30-A\prime 0}{A30-A0}\times 100\%$$

where A′0 and A′30 are the absorbance of the experimental group at 0 and 30 min; A0 and A30 are the absorbance of the blank control group at 0 and 30 min.

After collecting the cell lysate, the insoluble material was removed by centrifugation at 2000× *g* at 4 °C for 10 min and the supernatant was retrieved and stored at –20 °C or lower for subsequent use. The ROS and GSH content in B16 cells were determined according to the kit instructions of Nanjing Jiancheng Technology Co., Ltd.; the content of cyclic adenosine monophosphate (cAMP) was measured in accordance with the directions of the ELK Biotechnology Co., Ltd. kit.

### 2.6. Western Blot

Western blot was performed on whole cell lysates using the technique of Pazilaiti Ainiwaer et al. as a reference, with appropriate improvements [15]. B16 melanoma cells (1 × 10^5^ cells/well) were treated with 0.01, 0.05, 0.1 and 0.5 mg/mL of SCP1s for 48 h to determine the amount of MC1R, MITF, TYR, TRP-1, TRP-2, CREB and p-CREB. Cellular proteins were then extracted with lysis buffer and centrifuged at 16,000× *g* for 5 min at 4 °C and total concentration of protein was determined using the BCA protein assay reagent. SDS-PAGE was used to separate the samples into equal amounts of proteins, which were then transferred to PVDF membranes. The membranes were blocked with BSA for 1 h before being incubated with primary antibody overnight at 4 °C with continuous shaking. The membranes were washed with TBST and then incubated with the corresponding HRP conjugated secondary antibodies for 30 min at room temperature (25 °C). After washing the membranes, ECL was used to carry out chemiluminescent detection, the films were scanned and archived using a scanner (LiDE110, Canon Co., Ltd., Tokyo, Japan) and the optical density values of the target bands were assessed using the AlphaEaseFC (Alpha Inc., San Leandro, CA, USA) software processing system.

### 2.7. Real-Time Fluorescence Quantitative PCR (RT-PCR)

To determine the effect of SCPs1 on the expression of melanogenic genes, the method by YiChen Wang et al. was used as a reference [2], with slight modifications, and analysis was carried out using RT-PCR. B16 cells were subjected to treatment for 48 h with 0.01, 0.05, 0.1 and 0.5 mg/mL SCP1s, and the TYR, TRP-1, TRP-2, MITF and MC1R mRNA levels were examined. Total RNA extracts and reverse transcription were carried out in accordance with the manufacturer’s recommendations, and PCR was performed to detect the expression of relevant genes under the following conditions: the cDNA template was denatured at 95 °C for 30 min and the reaction was performed for 40 cycles of 95 °C for 10 s, 58 °C for 30 s, and 72 °C for 30 s. β-actin was used as an internal control. Primers were as follows (Table 1).

### 2.8. Data Analysis

Graphing and statistical analysis of data were performed using GraphPad Prism 8.0 (GraphPad Prism Inc., San Diego, CA, USA). Significance tests were performed using analysis of one-way ANOVA, followed by the post hoc Tukey’s test for multiple mean comparisons (**** *p* < 0.0001, *** *p* < 0.001, ** *p* < 0.01 and * *p* < 0.05). All experiments were repeated at least three times and data are presented as the mean ± standard deviation (mean ± SD).

## 3. Results and Discussion

### 3.1. Effect of SCPs1 on the Viability of B16 Cells

**Figure 1:**
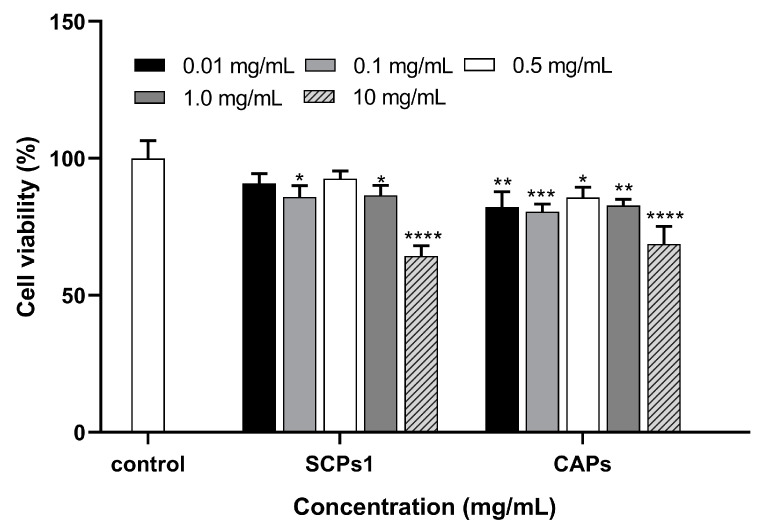
Effects of different concentrations of SCPs1 and CAPs on the viability of B16 cells for 48 h. **** *p* < 0.0001, *** *p* < 0.001, ** *p* < 0.01 and * *p* < 0.05 indicate that there is significant difference between different treatment methods and the control group.

As Figure 1 shows, cell viability was significantly (*p* < 0.0001–0.05) inhibited in the CAPs group compared to the control group but not significantly (*p* > 0.05) different between the 0.01 and 0.5 mg/mL SCPs1 groups. When the added concentration was 10 mg/mL, the cell viability of SCPs1 and CAPs groups significantly (*p <* 0.0001) decreased to 64.33% and 68.67%, respectively, exceeding the optimum safe concentration [16,17]. Therefore, the concentration range of 0.01–1.0 mg/mL (cell viability > 80%) was selected for the next step of melanin content assay.

### 3.2. Inhibitory Effect of SCPs1 on Melanin

**Figure 2:**
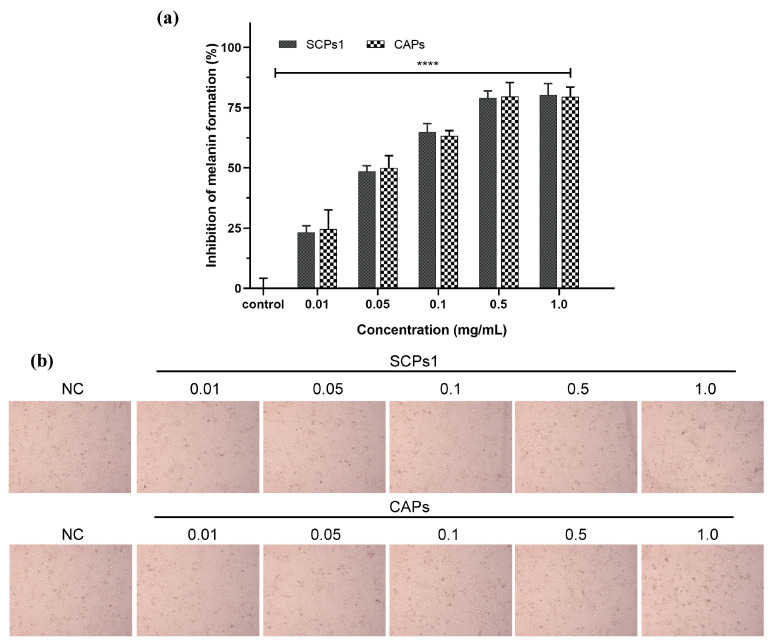
(**a**) Effects of different concentrations of SCPs1 and CAPs on melanin content in B16 cells. **** *p* < 0.0001 indicate that there is significant difference between different treatment methods and the control group. (**b**) Morphological observation of melanin in B16 cells with different concentrations of SCPs1 and CAPs.

The results in Figure 2a show that SCPs1 and CAPs significantly inhibited melanogenesis in B16 cells in the range of 0.01–1.0 mg/mL (*p* < 0.0001), with a dose-dependent increase in melanin content inhibitory rate, and there was little difference in the inhibitory effects between 0.5 and 1 mg/mL added concentrations. There was no significant difference between SCPs1 and CAPs at the same concentration (*p* > 0.05). Figure 2b is consistent with the results in Figure 2a. Compared with the group of 0.01 mg/mL, the cell count of 1 mg/mL group was obviously reduced, and the decrease in the B16 cell led to a decrease in melanin. Xinyao Ju et al. studied the effect of tilapia scale peptides on the melanin content of B16 cells, and the results showed that the melanin inhibitory rate was 47.85% when the added concentration was 0.05 mg/mL, which was similar to the results obtained in this study [18]. Yuqiu Wang et al. showed that silk fibroin could effectively reduce the melanin content of B16 cells by about 50% after adding a concentration of 1 mg/mL [19]. In this study, the melanin inhibitory rate was as high as 80.24% when SCPs1 was added at 1 mg/mL. This may be due to the decrease in the molecular weight of collagen peptides, resulting in more amino acid exposure, which is more conducive to the inhibition of melanogenesis in B16 cells [20]. In addition, the melanin inhibitory effect was also related to the collagen peptide source, amino acid composition and peptide sequence [21]. Zizi Hu et al. showed that the hydrophobic amino acids contained in the collagen peptide of grass carp scales could effectively inhibit the production of melanin, which was related to the tendency of pepsin to lyse hydrophobic amino acids [22]. This further indicated the possibility that SCPs1 had more hydrophobic amino acids. Yejun Deng et al. also showed that the hydrophobic amino acids contained in the peptide sequence inhibited melanogenesis to some extent [23]. Hu Hou et al. showed that macromolecular weight collagen peptides have a stronger inhibitory effect on melanogenesis [24], which may also be the reason why macromolecule SCPs1 has a higher inhibitory effect on melanogenesis.

### 3.3. Effect of SCPs1 on TYR Activity and ROS, GSH and cAMP Content

**Figure 3:**
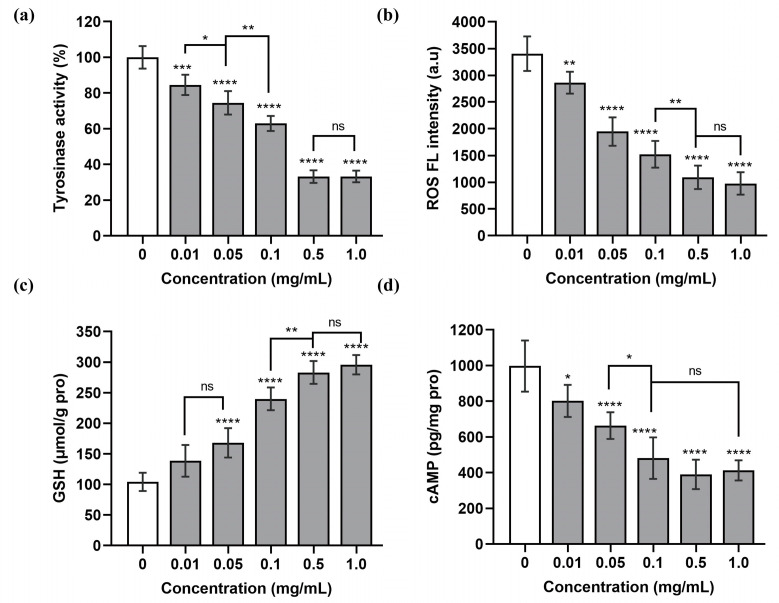
Effects of different concentrations of SCPs1 on (**a**) tyrosinase (TYR) activity, (**b**) reactive oxygen species (ROS), (**c**) glutathione (GSH) and (**d**) cyclic adenosine monophosphate (cAMP) content in B16 cells. **** *p* < 0.0001, *** *p* < 0.001, ** *p* < 0.01 and * *p* < 0.05 indicate that there is significant difference between different treatment methods and the control group; ns indicates that there is no significant difference between different treatments.

**Figure 4:**
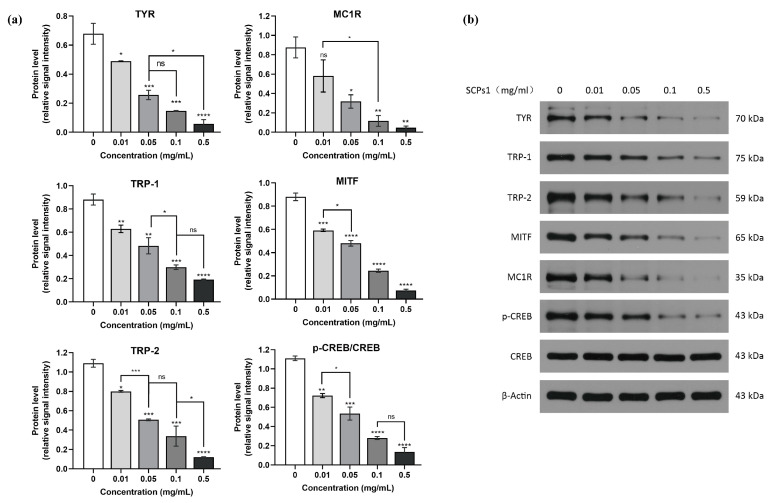
Effects of different concentrations of SCPs1 on the protein expression of cAMP-CREB signaling pathway in B16 cells. (**a**) Densitometry graphs of the Western blot analyses. (**b**) Protein levels of TYR, TRP-1, TRP-2, MITF, MC1R, *p*-CREB and CREB were examined by Western blot. **** *p* < 0.0001, *** *p* < 0.001, ** *p* < 0.01 and * *p* < 0.05 indicate that there is significant difference between different treatment methods and the control group; ns indicates that there is no significant difference between different treatments.

Tyrosinase is a key enzyme involved in intracellular melanogenesis, and inhibition of TYR activity can reduce melanogenesis. The results shown in Figure 3a,b,d indicate that, with the increase in SCPs1 concentration, the TYR activity and ROS and cAMP content of B16 cells showed a significant decrease, with the lowest decreases reaching 33.19%, 976.73 a.u. and 390.27 pg/mg pro, respectively. According to Figure 3c, the concentration of SCPs1 showed a positive correlation with the GSH content and, when the added concentration was 1 mg/mL, the GSH content was 2.83 times higher than that of the control group. In the process of melanogenesis, TYR requires ROS to catalyze the production of L-DOPA to promote melanin production [23,25]. Oxidative stress caused by UV radiation increases the intracellular level of ROS, resulting in an increase in TYR activity [26]. The results shown in Figure 3a,b also indicate reduced tyrosine activity, possibly due to the ROS scavenging ability of SCPs1. GSH has a scavenging effect on oxygen free radicals and is an effective antioxidant. Related research has shown that collagen peptides can promote the production of GSH and thus achieve ROS scavenging effect [27,28], which further explains the reduced tyrosinase activity observed in this study. With cAMP being a key messenger of the cAMP-CREB signaling pathway [29], the determination of cAMP content can preliminarily determine whether SCPs1 inhibits melanogenesis via this pathway. Figure 3d shows that cAMP content decreased with increasing SCPs1 concentration, indicating that SCP1s may be involved in the regulation of melanogenesis through the cAMP-CREB signaling pathway (shown in Figure 4). In Figure, the indicators were not significantly different (*p* > 0.05) between SCPs1 at an added concentration of 0.5 versus 1 mg/mL; hence, 0.01–0.5 mg/mL concentrations were selected for further exploration of cAMP-CREB signaling-pathway-related proteins.

### 3.4. Downregulation of cAMP-CREB Signaling Pathway Protein Expression by SCPs1

**Figure 5:**
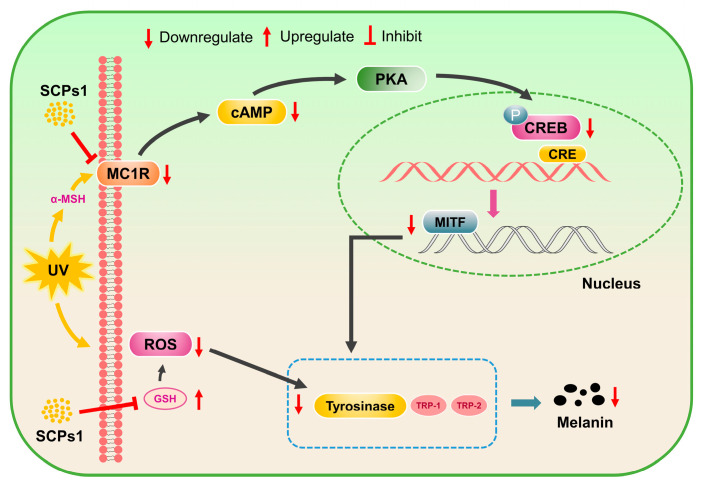
Mechanism of SCPs1 inhibiting melanogenesis in B16 mouse melanoma cells. The effects of cAMP-CREB pathway, GSH and ROS on melanogenesis were studied.

Changes in the relevant proteins in the cAMP-CREB pathway may be related to tyrosinase activity as well as melanogenesis [30]. To investigate the effect of SCPs1 on the relevant proteins in the cAMP-CREB pathway, such as TYR, TRP-1, MITF and MC1R, protein levels were examined via Western blot. Figure 4 shows that the protein expression of all three enzymes, TYR, TRP-1 and TPR-2, decreased with the increase in SCPs1 concentration, which is similar to what is shown in Figure 3a. From the changes in protein levels of MITF shown in Figure 4, it is evident that MITF levels decreased in a concentration-dependent manner for SCPs1 and the strongest inhibitory effect was observed when the added concentration was 0.5 mg/mL (*p* < 0.0001). The above results suggest that SCPs1 may downregulate MITF expression, thereby inhibiting the activity of TYR, TRP-1 and TPR-2. MC1R and CREB have been implicated in the regulation of MITF levels [31]. Hsiu-Mei Chiang et al. showed that *Rhodiola rosea* extract significantly inhibited the expression of MC1R and phosphorylation of CREB, thus downregulating the expression of MITF- and TYR-related proteins [32]. Therefore, we further investigated whether MITF was similarly regulated by both MC1R and CREB upstream pathway proteins after SCPs1 treatment. The results showed that the higher the added concentration of SCPs1, the lower the level of MC1R, with MC1R significantly reduced by 94.52% compared to the control group at an SCPs1 concentration of 0.5 mg/mL (*p* < 0.01). Similarly, SCPs1 significantly inhibited the phosphorylation of CREB, leading to a reduction in the level of MITF. Taken together, SCPs1 can inhibit melanogenesis by downregulating the relevant proteins in the cAMP-CREB pathway (show in Figure 5).

### 3.5. Effect of SCPs1 on the Transcription Levels of Genes in the cAMP-CREB Signaling Pathway

**Figure 6:**
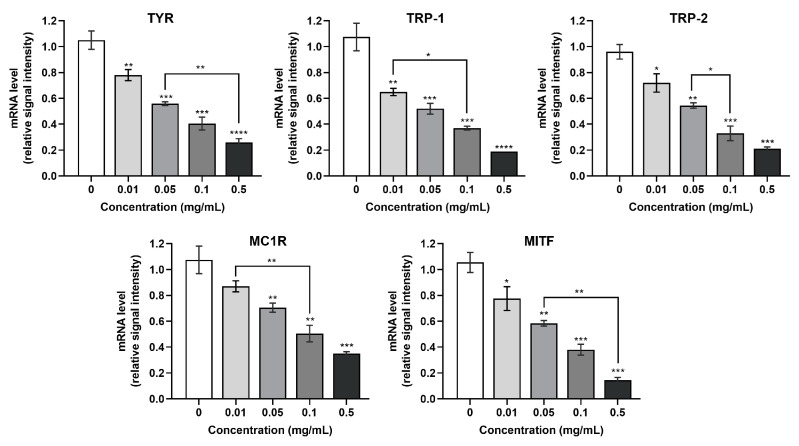
Effects of different concentrations of SCPs1 on transcription levels of cAMP-CREB signaling pathway genes in B16 cells. RT-PCR was used to detect the mRNA expression levels of TYR, TRP-1, TRP-2, MITF and MC1R in B16 cells. **** *p* < 0.0001, *** *p* < 0.001, ** *p* < 0.01 and * *p* < 0.05 indicate that there is significant difference between different treatment methods and the control group.

In addition to investigating the effect of SCPs1 on melanogenesis at the protein level, we further determined whether SCPs1 affects the transcription levels of genes in the melanogenesis pathway using RT-PCR. As Figure 6 shows, the mRNA transcription levels of TYR, TRP-1 and TRP-2 were significantly decreased in a dose-dependent manner after treatment of B16 cells with 0.01–0.05 mg/mL of SCPs1. The mRNA transcription levels of MITF and MC1R similarly decreased significantly with increasing added concentrations of SCPs1. RT-PCR could explain whether SCPs1 regulates melanogenic genes through transcription or post-transcription. Shuting Hu et al. showed that resveratrol regulated TRP-1 and TRP-2 at the transcriptional level in B16 cells, whereas MITF was observed at the post-transcriptional level as mRNA levels were not significant [33]. In this study, the mRNA level results corroborate with the results shown in Figure 6, indicating that SCPs1 first downregulated genes associated with the cAMP-CREB pathway at the transcriptional level, thus leading to a decrease in protein expression. In conclusion, SCPs1 inhibited melanogenesis by downregulating the expression of genes associated with the cAMP-CREB pathway at the mRNA level.

## 4. Conclusions

In this study, using SCPs1 as the experimental target, we investigated the inhibitory effect of SCPs1 on melanogenesis in B16 cells by measuring cell viability; melanin content; tyrosinase activity; and ROS, GSH and cAMP content and examining the expression levels of proteins and transcription levels of genes that are associated with the cAMP-CREB signaling pathway. The results showed that SCPs1 may affect melanogenesis by increasing GSH content, decreasing ROS and cAMP content and inhibiting tyrosinase activity. In addition, SCPs1 may downregulate the expression of genes related to the cAMP-CREB signaling pathway at the transcriptional level, leading to downregulation of protein expression, thus inhibiting melanogenesis. The current study only addressed the effect of SCPs1 on the cAMP-CREB signaling pathway and further investigations should be carried out to determine whether there are other existing pathways that are also involved in melanogenesis.

## Figures and Tables

**Table 1:** Primer sequences for real-time fluorescence quantitative PCR (RT-PCR).

Primers Names	Gene Accession Numbers	Primers Sequences (5’-3’)	
M-actin	NM_007393.5	sense	CTGAGAGGGAAATCGTGCGT
		antisense	CCACAGGATTCCATACCCAAGA
M-tyr	NM_011661	sense	CAAATTGTACAGAGAAGCGAGTCTT
		antisense	GGATGACATAGACTGAGCTGATAGT
M-trp-1	NM_031202	sense	CTCACAGTCAGGAGAAATCTTCTAG
		antisense	CAGTATGTCTTCTAACCTCCTTGTG
M-trp-2	NM_010024	sense	CACAGGAAACTTTGCTGGTTATAAT
		antisense	GATCACGTAGTCTGGATGGATACTC
M-mtif	NM_001113198	sense	CAAGTACCACATACAGCAAGCTC
		antisense	CTTATAAAATGCCTCTTTTTCACAG
M-mc1r	NM_008559	sense	GCTGGAGACTACTATCATCCTGCT
		antisense	GAGATGTAGCGGTCTATAGCAATG

## Data Availability

The data shown in this study are contained within the article.

## References

[1] Dzhussoeva E.V., Gorkun A.A., Zurina I.M., Kosheleva N.V., Kolokol’tsova T.D., Saburina I.N. (2020). Influence of Fucoxanthin on Proliferative Activity of Human Melanocyte Culture. Bull. Exp. Biol. Med..

[2] Wang Y.-C., Haung X.-Y., Chiu C.-C., Lin M.-Y., Lin W.-H., Chang W.-T., Tseng C.-C., Wang H.-M.D. (2019). Inhibitions of melanogenesis via Phyllanthus emblica fruit extract powder in B16F10 cells. Food Biosci..

[3] Park C.H., Kim G., Lee Y., Kim H., Song M.J., Lee D.H., Chung J.H. (2021). A natural compound harmine decreases melanin synthesis through regulation of the DYRK1A/NFATC3 pathway. J. Dermatol. Sci..

[4] Hu Y., Zhou Y., Hu X., Chen Q., Shi Y., Zhuang J., Wang Q. (2021). Cefotaxime sodium inhibited melanogenesis in B16F10 cells by cAMP/PKA/CREB pathways. Process Biochem..

[5] Lee H.R., Jung J.M., Seo J.Y., Chang S.E., Song Y. (2021). Anti-melanogenic property of ginsenoside Rf from Panax ginseng via inhibition of CREB/MITF pathway in melanocytes and ex vivo human skin. J. Ginseng Res..

[6] An X., Lv J., Wang F. (2022). Pterostilbene inhibits melanogenesis, melanocyte dendricity and melanosome transport through cAMP/PKA/CREB pathway. Eur. J. Pharmacol..

[7] Zhang X., Li J., Li Y., Liu Z., Lin Y., Huang J.A. (2020). Anti-melanogenic effects of epigallocatechin-3-gallate (EGCG), epicatechin-3-gallate (ECG) and gallocatechin-3-gallate (GCG) via down-regulation of cAMP/CREB/MITF signaling pathway in B16F10 melanoma cells. Fitoterapia.

[8] Liu Y.Y., Su X.R., Liu S.S., Yang S.S., Jiang C.Y., Zhang Y., Zhang S. (2017). Zebrafish phosvitin-derived peptide Pt5 inhibits melanogenesis via cAMP pathway. Fish Physiol. Biochem..

[9] Faralizadeh S., Rahimabadi E.Z., Bahrami S.H., Hasannia S. (2021). Extraction, characterization and biocompatibility evaluation of silver carp (*Hypophthalmichthys molitrix*) skin collagen. Sustain. Chem. Pharm..

[10] Chen X., Li X., Yang F., Wu J., Huang D., Huang J., Wang S. (2022). Effects and mechanism of antifreeze peptides from silver carp scales on the freeze-thaw stability of frozen surimi. Food Chem..

[11] Shen X., Li T., Li X., Wang F., Liu Y., Wu J. (2022). Dual cryoprotective and antioxidant effects of silver carp (*Hypophthalmichthys molitrix*) protein hydrolysates on unwashed surimi stored at conventional and ultra-low frozen temperatures. LWT Food Sci. Technol..

[12] Liu S.C., Sheu M.L., Tsai Y.C., Lin Y.C., Chang C.W., Lai D.W. (2022). Attenuation of in vitro and in vivo melanin synthesis using a Chinese herbal medicine through the inhibition of tyrosinase activity. Phytomedicine.

[13] Zeng H.J., Li Q.Y., Ma J., Yang R., Qu L.B. (2021). A comparative study on the effects of resveratrol and oxyresveratrol against tyrosinase activity and their inhibitory mechanism. Spectrochim. Acta. A Mol. Biomol. Spectrosc..

[14] Ando H., Itoh A., Mishima Y., Ichihashi M. (1995). Correlation between the number of melanosomes, tyrosinase mRNA levels, and tyrosinase activity in cultured murine melanoma cells in response to various melanogenesis regulatory agents. J. Cell. Physiol..

[15] Ainiwaer P., Nueraihemaiti M., Li Z., Zang D., Jiang L., Li Y., Aisa H.A. (2022). Chemical constituents of Ruta graveolens L. and their melanogenic effects and action mechanism. Fitoterapia.

[16] Kim K., Jeong H.I., Yang I., Nam S.J., Lim K.M. (2021). Acremonidin E produced by Penicillium sp. SNF123, a fungal endophyte of Panax ginseng, has antimelanogenic activities. J. Ginseng Res..

[17] Yu Z.-Y., Xu K., Wang X., Wen Y.-T., Wang L.-J., Huang D.-Q., Chen X.-X., Chai W.-M. (2022). Punicalagin as a novel tyrosinase and melanin inhibitor: Inhibitory activity and mechanism. LWT Food Sci. Technol..

[18] Ju X., Cheng S., Li H., Xu X., Wang Z., Du M. (2022). Tyrosinase inhibitory effects of the peptides from fish scale with the metal copper ions chelating ability. Food Chem..

[19] Wang Y., Duan T., Hong M., Zhou Y., Huang H., Xiao X., Zheng J., Zhou H., Lu Z. (2021). Quantitative proteomic analysis uncovers inhibition of melanin synthesis by silk fibroin via MITF/tyrosinase axis in B16 melanoma cells. Life Sci..

[20] Park S.H., Jo Y.-J. (2019). Static hydrothermal processing and fractionation for production of a collagen peptide with anti-oxidative and anti-aging properties. Process Biochem..

[21] Li C., Fu Y., Dai H., Wang Q., Gao R., Zhang Y. (2022). Recent progress in preventive effect of collagen peptides on photoaging skin and action mechanism. Food Sci. Hum. Well..

[22] Hu Z.-Z., Ma T.-X., Sha X.-M., Zhang L., Tu Z.-C. (2022). Improving tyrosinase inhibitory activity of grass carp fish scale gelatin hydrolysate by gastrointestinal digestion: Purification, identification and action mechanism. LWT Food Sci. Technol..

[23] Deng Y., Huang L., Zhang C., Xie P., Cheng J., Wang X., Liu L. (2020). Skin-care functions of peptides prepared from Chinese quince seed protein: Sequences analysis, tyrosinase inhibition and molecular docking study. Ind. Crop. Prod..

[24] Hou H.U., Zhao X.U.E., Li B., Zhang Z., Zhuang Y. (2011). Inhibition of Melanogenic Activity by Gelatin and Polypeptides from Pacific Cod Skin in B16 Melanoma Cells. J. Food Biochem..

[25] Castro-Jácome T.P., Alcántara-Quintana L.E., Montalvo-González E., Chacón-López A., Kalixto-Sánchez M.A., del Pilar Rivera M., López-García U.M., Tovar-Pérez E.G. (2021). Skin-protective properties of peptide extracts produced from white sorghum grain kafirins. Ind. Crop. Prod..

[26] Gui M., Du J., Guo J., Xiao B., Yang W., Li M. (2014). Aqueous Extract of Chrysanthemum morifolium (Ju Hua) Enhances the Antimelanogenic and Antioxidative Activities of the Mixture of Soy Peptide and Collagen Peptide. J. Tradit. Complement. Med..

[27] Xiang Z., Xue Q., Gao P., Yu H., Wu M., Zhao Z., Li Y., Wang S., Zhang J., Dai L. (2023). Antioxidant peptides from edible aquatic animals: Preparation method, mechanism of action, and structure-activity relationships. Food Chem..

[28] Zhang C., Du B., Song Z., Deng G., Shi Y., Li T., Huang Y. (2023). Antioxidant activity analysis of collagen peptide-magnesium chelate. Polym. Test..

[29] Aguilar-Toala J.E., Hernandez-Mendoza A., Gonzalez-Cordova A.F., Vallejo-Cordoba B., Liceaga A.M. (2019). Potential role of natural bioactive peptides for development of cosmeceutical skin products. Peptides.

[30] Guo L., Yin Z., Wen L., Xin J., Gao X., Zheng X. (2019). Flower extracts from Paeonia decomposita and Paeonia ostii inhibit melanin synthesis via cAMP-CREB-associated melanogenesis signaling pathways in murine B16 melanoma cells. J. Food Biochem..

[31] Jeon N.-J., Kim Y.-S., Kim E.-K., Dong X., Lee J.-W., Park J.-S., Shin W.-B., Moon S.-H., Jeon B.-T., Park P.-J. (2018). Inhibitory effect of carvacrol on melanin synthesis via suppression of tyrosinase expression. J. Funct. Foods.

[32] Chiang H.M., Chien Y.C., Wu C.H., Kuo Y.H., Wu W.C., Pan Y.Y., Su Y.H., Wen K.C. (2014). Hydroalcoholic extract of *Rhodiola rosea* L. (*Crassulaceae*) and its hydrolysate inhibit melanogenesis in B16F0 cells by regulating the CREB/MITF/tyrosinase pathway. Food Chem. Toxicol..

[33] Hu S., Zheng Z., Zhang X., Chen F., Wang M. (2015). Oxyresveratrol and trans-dihydromorin from the twigs of Cudrania tricuspidata as hypopigmenting agents against melanogenesis. J. Funct. Foods.

